# Inter-organizational collaboration at the local level—models and experiences

**Published:** 2012-09-04

**Authors:** Runo Axelsson

**Affiliations:** Sahlgrenska Academy, The Medical Faculty of the University of Gothenburg, Sweden

**Keywords:** integration, collaboration, organizational models, interorganizational

## Abstract

Integration and collaboration has been an important strategy for development of the health and welfare system in Sweden since the 1960s. This development has been intensified during the 1990s and the first decade of the 2000s. This key note speech will focus on the development of interorganizational collaboration between health care, social service and other welfare services on the local level of the Swedish society. There will be examples from care of the elderly, social psychiatric care and vocational rehabilitation. Different organizational models will be presented and experiences of barriers as well as facilitating factors will be discussed.

Powerpoint presentation available at http://www.integratedcare.org at congresses – San Marino – programme.

